# Additive Manufacturing Applications in Biosensors Technologies

**DOI:** 10.3390/bios14020060

**Published:** 2024-01-23

**Authors:** Abraham Abbey Paul, Adedamola D. Aladese, Robert S. Marks

**Affiliations:** 1 Avram and Stella Goldstein-Goren Department of Biotechnology Engineering, Ben-Gurion University of the Negev, Be’er Sheva 84105, Israel; paulab@post.bgu.ac.il; 2 Department of Physics and Material Science, University of Memphis, Memphis, TN 38152, USA; daladese@memphis.edu; 3 Ilse Katz Centre for Nanoscale Science and Technology, Ben-Gurion University of the Negev, Be’er Sheva 84105, Israel

**Keywords:** bioinks, polymers, 3D (bio)printing, biosensors, additive manufacturing

## Abstract

Three-dimensional (3D) printing technology, also known as additive manufacturing (AM), has emerged as an attractive state-of-the-art tool for precisely fabricating functional materials with complex geometries, championing several advancements in tissue engineering, regenerative medicine, and therapeutics. However, this technology has an untapped potential for biotechnological applications, such as sensor and biosensor development. By exploring these avenues, the scope of 3D printing technology can be expanded and pave the way for groundbreaking innovations in the biotechnology field. Indeed, new printing materials and printers would offer new possibilities for seamlessly incorporating biological functionalities within the growing 3D scaffolds. Herein, we review the additive manufacturing applications in biosensor technologies with a particular emphasis on extrusion-based 3D printing modalities. We highlight the application of natural, synthetic, and composite biomaterials as 3D-printed soft hydrogels. Emphasis is placed on the approach by which the sensing molecules are introduced during the fabrication process. Finally, future perspectives are provided.

## 1. Introduction

There has been a tremendous advancement in three-dimensional (3D) printing technologies for precisely fabricating functionalized biological constructs with complex geometries. Three-dimensional (3D) printing, also known as additive manufacturing (AM), has emerged as a promising technique in tissue engineering applications by precisely depositing cells and biomaterials layer-by-layer. AM involves computer-aided designing and prototyping for the layer-by-layer manufacturing of 3D objects using a broad range of printing materials referred to as ‘inks’. Similarly, 3D bioprinting is a type of additive manufacturing (AM) that involves extruding living cell-laden hydrogel suspensions, or bioinks, layer-by-layer through a nozzle, thus forming functional 3D structures with predesigned porous structures. A significant advantage of AM is the possibility to exert precise control over the microarchitecture of the printed structure by optimizing the mechanical, physical, and biological properties of inks and cell distribution [1]. Recent advances in 3D printing technologies and the ability to print biomaterials and functional entities simultaneously have opened new and rapidly growing opportunities in tissue engineering and biomedical fields.

Three-dimensional (3D) printing technologies have a high prospect of impacting the biosensor community near the sensor prototyping and sensing layer organization levels [2]. Realizing scaffolds with integrated sensor molecules could provide a viable route for applying spatiotemporal sensing capabilities to emerging 3D cell culturing environments. Currently, 3D printing technology is dominated by biomedical applications such as tissue engineering and regenerative medicine, and fewer studies have extended these technologies into biotechnological applications in sensors and biosensors development [3]. The incorporation of sensor or biosensor units into the 3D printing processes would allow a non-invasive spatial-temporal approach to monitoring cells’ microenvironment during culturing and serve as a multipurpose platform for the development of in vitro models for studying diseases and drug screening to decrease the use and sacrifice of animals [4].

To begin with, this article briefly reviews the current 3D printing technologies and the different printing materials. More importantly, recent research on the applications of 3D printing in biosensors was reviewed with an emphasis on fabrication methods and printing materials. Readers would be brought to speed on the current state of 3D printing applications in sensors and biosensors and highlighted the current challenges and future development opportunities in 3D printing-based biosensor technologies.

## 2. Three-Dimensional (3D) Printing and Sensors, in Retrospect

The miniaturizing of devices has aided the development of several analytical techniques, especially in biomedicine, chemistry, and engineering [5]. The focus is to make most systems become as small as possible [6]. This also applies to sensors as in vivo and in vitro monitors of biological signals. The breakthroughs in manufacturing microsystems further paved the way for biomedical sensors, which are classified under miniaturization, elastic nature, and design [7]. This implies a biosensor must be small, have an elastic modulus compatible with the human tissue, and must have the ability to be customized based on its functions. Available biomedical sensors in their small size are tactile sensors, microfluidic devices, diagnostic devices, biomarkers for diseases, and microelectrode-based electronic probes. The complexity of making micro and nanoscale devices started with etching on glass, which was later replaced with a silicon (Si) wafer [8]. Known for its outstanding characteristics over glass, Si wafer lithography has grown to a larger scale. Soft lithography uses an elastomeric polymer called polydimethylsiloxane (PDMS) to fabricate devices for various applications [9,10,11]. In contrast to glass and silicon, PDMS has a low elastic modulus (300–500 Kpa), high biocompatibility, optical transparency, and adaptable gas permeability [12,13], making it one of the best-fit materials as a biosensor [7]. The limitations of soft lithography include but are not limited to sophisticated processes (combined with photolithography), multiple stages, high professional experience, incompatibility with some reagents, and susceptibility to swelling while in contact with organic solvents [14]. Also, making a 3D structure is a significant setback in photo-soft lithography. A possible way is to stack several layers of 2D devices to form a 3D device [15]. Unfortunately, this approach is time-consuming and unreliable. The inception of 3D printing brought about a significant milestone in several fields and applications of microsystems, including microfluidics, biosensors, and biomedicine [16,17,18]. Three-dimensional (3D) printing, also known as additive manufacturing (AM), involves the layer-by-layer formation of an object to form a 3D structure. This process relies on extruded, deposited, or solidified materials under certain conditions in making the required object [19]. The American Society for Testing and Materials (ASTM) classified AM techniques based on their mode of operation into seven categories, which include powder bed fusion (PBF), vat polymerization, extrusion, material jetting, binder jetting, sheet lamination, and directed energy deposition (DED) [20].

## 3. An Overview of 3D Printing Technologies

**Figure 1:**
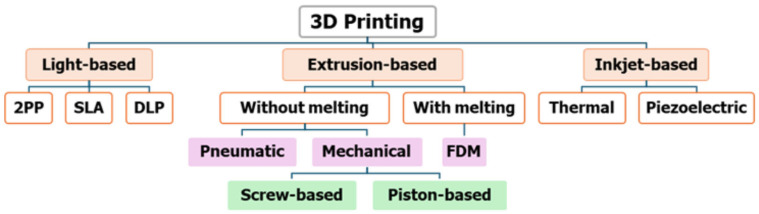
Three-dimensional (3D) Printing Technologies. SLA, stereolithography; 2PP, two-photon polymerization; digital light processing adapted from [21], Copyright (2021), with permission from Elsevier.

Additive manufacturing involves computer-aided design (CAD) and computer-assisted manufacturing (CAM) to build complex 3D construct models. These are then converted to STL format that can be sliced into 2D layers bound together. The sliced components can then be printed layer-by-layer by an automated deposition of inks onto a substrate using a 3D printer [21,22]. Standard 3D printing methods include stereolithography-based, inkjet, extrusion, and laser-assisted 3D printing (Figure 1) [21].

### 3.1. Different 3D Printing Methods and Materials

#### 3.1.1. Vat Photopolymerization

Vat photopolymerization is a popular 3D printing technique that utilizes ultraviolet (UV) light to solidify the liquid light-curable resin, forming chains between molecules and crosslinking the resin [23]. It is recognized for its capability to create complex structures with fine details and high surface quality [24]. Here, liquid resins are polymerized under ultraviolet (UV) using particular wavelengths and printing conditions. The photosensitive resin is placed in a vat and cured within a few seconds, making it a rapid and accurate method compared to other AM processes [25]. This technique is divided into stereolithography (SLA), digital light processing (DLP), and two-photon polymerization (2PP) [26,27,28]. In SLA, the system is further subdivided into free-surface and constrained surface configurations, depending on the orientation of the light source for curing the resins. The constrained surface has proven more advantageous for several applications than the free-surface configuration. At first, the object’s height is not restricted by the size of the vat. Secondly, the curing time is faster, and less resin is wasted. A slight modification to the SLA is micro-stereolithography (µSLA) enhanced with a resolution on a micron level [29]. The DLP printer uses a similar operating procedure as in a SLA-constrained surface system, except that a digital projector is used to cure the photo resins instead of UV light. This system is faster than SLA printers, and uncured resins can always be reused. The 2PP system was introduced to solve some of the shortcomings of SLA and DLP printers. Providing a higher accuracy, the 2PP system can achieve sub-100 nm resolution even though its printing speed is slow.

A movable 3D microfluidic biosensor chip was printed using micro stereolithography (SL), which contains a digital micromirror device (DMD) [30]. It consists of a torque-actuated pump and valve, rotary valve, and pushing valve to perform general colorimetric assays. Incorporating the 3D printed device helps reduce excess loss of reagents, allows for rapid on-site quantitative detection, and comes with easy operation protocol with low manufacturing cost. This technology, alongside the smartphone, was ascertained to perform an informative and broad quantitative analysis.

A fully automated microfluidic device was achieved via stereolithography printing. Fluidic valves and pumps were printed for cell culture applications [31]. Even though the ease of total fabrication was reduced, the performance of their device is still inferior to that of their PDMS counterparts. Still, there are prospects that their performances will be enhanced since SL has promising features in the future.

An SLA printer, an FDM printer, and an FDM printer aid the fabrication and characterization of a robust 3D-printed microfluidic analysis system. This device can be incorporated with FDA-approved clinical microdialysis probes to monitor human tissue metabolite levels [27] continuously.

#### 3.1.2. Powder Bed Fusion

This technique involves the layer-by-layer fusion of powdered material to create three-dimensional objects, and it is prevalent for its ability to produce small, precision-demanding, and diverse pieces [32]. This technology operates on powdered material (metal, polymer, ceramics, composites, etc.) that is joined point by point using a laser beam or electron beam as an energy source. In this concept, a roller spreads over a thin layer of powder with the energy source melting or sintering it [33,34]. This process repeats itself until the whole structure is fully formed. The outcome of the 3D structure depends on various factors such as powder quality, spot size beam, or laser power. The common types of PBF are selective laser sintering (SLS), laser power bed fusion (LPBF), and electron beam melting (EBM). The significant disadvantages of this technique are the post-processing requirement, difficulty in controlling porosity, and slow printing time. For the electrochemical detection of ascorbic and uric acids, stainless electrodes were printed using metal 3D printing technology. The resulting device performes excellently compared to the conventional method of glassy carbon electrodes in terms of sensitivity, selectivity, and reproducibility, and having a linearity range of 0.1–1 mM [35].

#### 3.1.3. Material Jetting

This ink and powder liquid extrusion method is like printing text and images. The printheads are similar to those in inkjet printers except that the ink here is deposited in microdroplets, forming a layer-by-layer formation of the 3D structure [36]. This non-contact technique offers an accurate print around edges, curves, and incline surfaces. Commonly used materials include photo resins, carbon nanotubes, and hydrogels. Examples include inkjet printing (IJP), Aerosol Jet printing (AJP), MultiJet printing (MJP), and Polyjet. In AJP, the droplets are aerosols, which are transported to the nozzle or orifice by a gas. An Objet Connex 350 Multi-material printer fabricated an electrochemical detection device. A microfluidic platform with a threaded receiving port that allows integration of different electrode materials for dopamine and nitric oxide detection and a device that collects adenosine triphosphate (ATP) while measuring the release stimulus of reduced oxygen concentration were 3D printed. This sensor could detect neurotransmitters and signaling molecules [37]. The results demonstrate the reproducibility and transferability of 3D printing as a fabrication technique for the sensing devices and electrodes. A wireless, stretchable, implantable biosystem was 3D printed using a nano-ink system. The technology allows for high-performance multi-layer printing of a capacitance flow sensor [38].

#### 3.1.4. Extrusion-Based System

Extrusion-based 3D printing involves the ink deposition onto a stage along the X and Y axes, causing the layer-by-layer fabrication of the construct to advance along the *Z* axis by moving the head up or down. Extrusion 3D printing is classified into two sub-groups: processes involving material melting and those without material melting [21]. A commonly used extrusion-based 3D printing based on material melting is fused deposition modeling (FDM). In FDM, a nozzle ejects a plastic or ink to print an object [39,40]. A key feature in the material melting approach is that the material temperature is kept at a temperature just greater than its melting point for easy extrusion through the nozzle to allow the filaments to fuse during printing and to solidify upon cooling. They offer several benefits, such as low cost, full automation, and a wide array of printing materials. The available materials used in the fabrication include ceramics, polyethylene terephthalate (PET), wax blends, thermoplastic elastomers (TPE), nylon, metals, polylactic acid (PLA), acrylonitrile butadiene styrene (ABS), and polycarbonate (PC), polyvinyl alcohol (PVA), and cyclic olefin copolymer (COC). Most of these materials are hard plastics with poor gas permeability, which are not fitted for sensors application. A DIW printer was reportedly used to fabricate an implantable biosensor for monitoring glutamate. Here, a nanocomposite ink was printed on thin-film polymer substrates. Using this technique, it was discovered that the sensor had high sensitivity, low detection limit, and can sense glutamate for in vivo applications [41]. A micro-extrusion 3D printer was used to fabricate microfluidic-based microelectrochemical systems containing piezoelectric sensors and actuators for acoustofluidic applications. This device showcases the effectiveness of additive manufacturing integration with piezoelectric transducers [42]. The extrusion-based manufacturing without material melting can be classified into pneumatic and mechanical (screw and piston-based). This approach is commonly employed in tissue engineering bioprinting soft hydrogels because of its mild fabrication condition. In pneumatic 3D printing, air or gas pressure is used to extrude the ink through the nozzle at controlled printing speed, flow rate, volume, and pressure. This approach has a limit of viscosity that can be extruded based on the pressure limit. The mechanical-based extrusion uses a moving pistol and rotating screw to drive the ink out of the nozzle with better spatial control and a broader range of viscosities than the counterpart pneumatic approach.

#### 3.1.5. Inkjet Printing

Inkjet-based 3D printing is becoming increasingly popular due to its potential applications in various fields, including wearable and textile electronics, microstructures, and additive manufacturing. The versatile technology has shown promise in printing small-scale 3D objects, functional structures, and multi-material structures [43,44]. As a result, it has become a valuable tool for modern manufacturing processes [45]. The goal of ink-based 3D printing is the development of printable inks with precise rheological properties such as viscosity, shear yield stress, and surface tension. These technological advancements in 3D printing have enabled the fabrication of numerous 3D sensors, opening up new avenues in scientific research. [46]. Inkjet-based 3D printing encompasses two main types: thermal and piezoelectric inkjet 3D printing. In thermal inkjet 3D printing, a heating element functions as a thin film resistor. When an electrical pulse is applied, it generates a high current that vaporizes the adjacent ink into bubbles, leading to an expansion of vapor bubbles in the ink reservoir [21]. This expansion creates pressure, ejecting the ink droplets out of the nozzle. On the other hand, piezoelectric inkjet 3D printing involves applying an external voltage to a piezoelectric transducer, resulting in a sudden change in the volume of the ink chamber [47]. A flexible cylindrical enzyme-electrode glucose microsensor was fabricated using rotated inkjet printing. This concept uses maskless direct writing for in situ fabrication and modification on micro cylindrical substrates. This device showed glucose detection of 0–570 mg dL^−1^, which agrees with the clinical standards. The rotated inkjet printing method was then proposed as a prospective tool for fabricating flexible bioelectronics on arbitrary shapes of substrates on a micro-scale [48].

All the components of an enzymatic glucose sensor were fully printed using inkjet and recyclable paper as substrates. The printed device was unique as it is a metal-free fabrication and can detect glucose in saliva with a shelf life of thirty-one (31) days. This sensor’s components, including the conducting polymer, were printed as a layer-by-layer assembly [49]. Further examples of ink-based 3D printing applications in sensors are reviewed elsewhere [46].

## 4. 3D (Bio)Printers and Printing Materials

Three-dimensional (3D) printers have become very inexpensive, and various open-source architectures are available, further increasing the accessibility to this technology [50]. Three-dimensional (3D) bioprinters are broadly categorized into four groups based on their working principles. The following are types of bioprinters commonly used: stereolithography, acoustic, microvalve, inkjet-based, extrusion-based, laser-assisted, and needle array bioprinters. The choice of bioprinter should align with the specific requirements of the target construct and bioink. To choose a bioprinter for a particular application, two factors are usually considered: the structural properties of the target construct and the printing material (bioinks). The commonly regarded structural properties of the target construct include its size, complexity, and the type of tissues or organs being printed. In contrast, the properties of the bio-inks that are usually considered are their viscosity, composition, and crosslinking mechanisms, which are influential in determining the most suitable bioprinting technology. In addition, printing speed, resolution, scalability, and compatibility with the desired biomaterials are also considered.

### 4.1. Bio-Inks

Bioinks are materials used for 3D bioprinting, composed of living cells alone or with a supportive hydrogel component. These bioinks are designed to enable the fabrication of porous hydrogels, providing control over printability, mechanical properties, and degradation characteristics, which are essential for the custom 3D fabrication of resilient, cellularized structures [51]. These bioinks are cell-encapsulating biomaterials used in the 3D printing process and must be friendly to both the printing process and 3D cell culture. Bioinks are also being designed to hold growth factors and exhibit thermo-responsive properties [52], which are critical for applications in bone regeneration and drug delivery [53], as well as the incorporation of sensor units [50]. The active materials in bioinks can serve as an extracellular matrix that helps provide adhesion to cells, the proliferation of cells, and cellular differentiation after bioprinting. The biomaterials employed in bioink formation should exhibit high biocompatibility, mechanical strength, rheological property, printability, and bioactivity. Based on their origin, bioinks can be natural, synthetic, or hybrid materials interacting with biological systems in 3D bioprinting [54,55]. The materials used in the 3D bioprinting are usually processed into 3D insoluble aqueous matrices called hydrogels.

**Figure 2:**
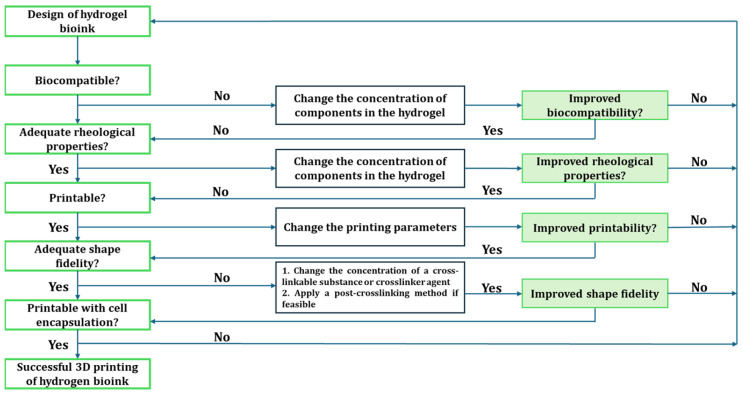
Flowchart for developing bioinks adapted with permission from [21], Copyright (2021), with permission from Elsevier. In additive manufacturing processes, the bioink properties and printing parameters undergo rounds of optimization steps. Some optimized parameters include bioink’s viscosity, gelation, printing speed, and temperature.

Hydrogels are 3D hydrophilic polymers that absorb and retain significant aqueous fluids without dissolving in the media. The hydrogel structure results when the hydrophilic domain of the polymeric networks becomes hydrated in an aqueous environment [56]. The term “network” in this context refers to the interconnected structure of the polymer chains and the presence of crosslinks, which is crucial for preventing the dissolution of polymer chains or segments into the surrounding aqueous phase [56]. They are widely used in fabricating 3D scaffolds for tissue engineering because of their viscosity, one of the essential factors for printability [57,58]. Other critical factors for printability are the rheological behaviors—the storage modulus (G′) and the loss modulus (G″) of the bioink, which can be readily tuned in many ways according to the printing needs [59]. Hydrogels with characteristically low viscosity and high loss modulus commonly exhibit limited rigidity to self-support the layer-by-layer deposition during the printing process, thereby necessitating the need for a crosslinking [56] step at a particular stage of the process—pre- or post-printing or simultaneously, during printing. A summary of how bio-inks are formulated for 3D bioprinting was put forward by [21], as shown in Figure 2.

Various techniques have been employed to create crosslinks to prepare hydrogels. Both chemical and physical methods have been used to form hydrogels. While the chemical method introduces a covalent bond between different polymer chains, the physical crosslinking is achieved via ionic interaction, and the latter is usually reversible by physical means. The work of Hennink and Nostrum [56] provides an extensive description of various hydrogel crosslinking methods and their mechanisms of action. The choice of crosslinking methods and the controllability of the crosslinking process during bioprinting is important for printability. A great deal of research effort is directed to the formulation of printing material that would offer high printing fidelity and structural integrity.

Technically, there are two broad categories of bioinks—low-viscosity and high-viscosity biomaterial inks. Low viscosity bioinks tend to collapse after extrusion [56] due to gravity, compromising the printing fidelity and thus requiring a technological effort for processing. Approaches like in situ crosslinking for ionically crosslink-able inks, UV exposure for the inks with photoreactive groups, and printing into sacrificial support baths [60] are the commonplace post-processing of the plotted low viscosity inks constructs [61]. High viscosity bioinks allow the direct fabrication of volumetric constructs in the air and can withstand structure collapse. Both high and low viscosities bioinks have advantages and disadvantages in AM, depending on the intended applications. For instance, a composite of alginate and gelatine has been employed in 3D printing, whereby gelatine provided the initial structural support to the construct before adding calcium chloride for alginate crosslinking.

### 4.2. Different (Bio)Materials Used in 3D Printing

**Figure 3:**
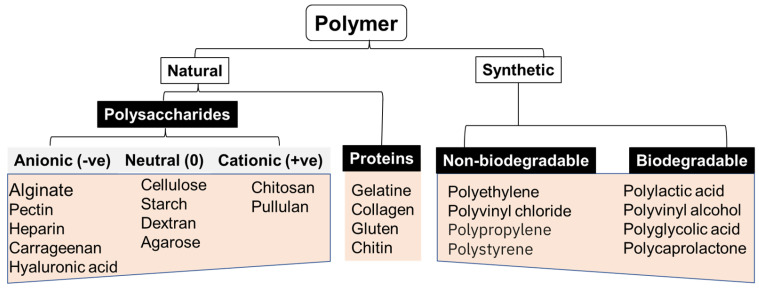
Classification of polymers based on their source.

New printing biomaterials and formulations have offered versatility to various 3D bioprinting applications. Additive fabrication techniques can work with multiple materials, but not all are suitable for all applications, including biosensing. Custom materials with tailorable properties that address the application’s needs must be designed for all of these techniques. Synthetic and naturally occurring polymers or a combination of both (semi-synthetic) (shown in Figure 3) are used in 3D printing depending on the intended applications and the desired physicochemical properties of the bioink [62].

The naturally occurring polymers in 3D printing include alginate, gelatine, chitosan, collagen, hyaluronic acid, and fibrinogen/fibrin, while synthetic materials include polyvinyl alcohol. These polymers have been used individually or as composites for bioprinting applications (Table 1).

## 5. Biosensors: An Overview

The demand to produce sensing devices rapidly and cost-effectively for medical diagnostics, environmental monitoring, and process industries is on the rise. In recent decades, research on biosensor technology has witnessed tremendous progress in response to the increasing spectrum of clinical and environmental analytes, necessitating the need for more rapid and affordable analytical tools. Modern and conventional analytical techniques can offer appreciable accurate and sensitive detection of clinical and environmental analytes. Still, most of these techniques are limited by cost, the need for trained personnel, and impracticability for onsite analysis [67]. Biosensors are seen as the tools of choice to match the rising need for diagnostics and monitoring, as they are inexpensive, seamless to construct, and, most importantly, can be miniaturized into portable formats.

**Figure 4:**
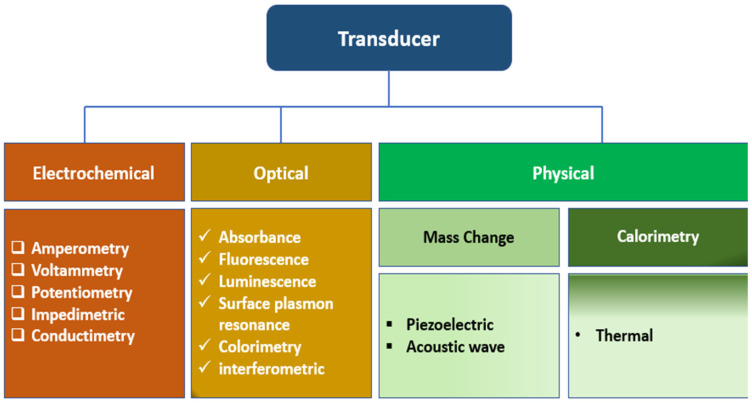
The classification of biosensors based on the types of transducers.

Biosensors are compact analytical devices incorporating a biological sensing element. Biosensors can detect biomolecules in a complex sample by converting the physical or chemical signal into an optical or electrical signal, which can be further processed to yield the analyte concentration, quantitative or semi-quantitatively [68]. Biosensors offer several advantages over conventional analytical methods, such as speed, ease of use, low cost, non-destructive properties, and on-site detection, making them indispensable tools in various fields [69]. They comprise the biological recognition element (BRE), signal transducers, and display units. Biosensors can be classified based on the type of biorecognition element or the nature of transducers used in the device’s development. Based on the kind of biorecognition, biosensors can be classified into catalytic (such as enzymes and catalytically active polynucleotides—DNAzymes) and affinity types such as nucleic acid biosensors and immunosensors). In some whole-cell biosensors, living cells and microorganisms can act as recognition elements (bioreporters) responding to trigger molecules such as inducers) by expressing specific (indicator) genes. The BRE, such as hormones, receptors, antigens/antibodies, enzymes, living cells, nucleic acids, carbohydrates, and tissues, specifically recognize the analytes via catalysis or affinity interactions, which can be further processed to quantify the amount of the analyte in a given sample [70]. Enzyme-based biosensors use specific biochemical recognition and high-efficiency catalysis, leveraging high catalytical power to produce a biosensor with high selectivity and ultra-low detection limits. Other biorecognition molecules such as antigen/antibody, nucleic acid/complementary sequences, and protein/receptor interactions employ a high affinity specific binding interaction as molecular recognition, forming a stable complex. Biosensors can also be classified as optical [71], electrochemical (label-based or label-free), mechanical [72], and conductometric [73] biosensors. Also, based on the signal transducers, biosensors can be classified as thermal, electrochemical, piezoelectric, magnetic, optical, mechanical, or radioactive sensors (Figure 4).

### 5.1. Three Categories of Biosensors Based on the Types of Transducers

#### 5.1.1. Optical Biosensors in Additive Manufacturing Processes

Optical biosensors are widely adapted analytical techniques for the real-time detection of analytes in clinical and environmental samples owing to their high sensitivity and selectivity, ease of deployment, and cost-effectiveness. In this type of biosensor set-up, the information is transduced in the form of photons as absorbance, reflectance, luminescence, or fluorescence emissions over the ultraviolet (UV), visible, or near-infrared regions of the electromagnetic spectrum, as shown in Figure 4. By far, fluorescence is the most applied optical detection method and comes in different measurement formats, including fluorescence intensity, quenching efficiency, anisotropy, and decay time, among others.

**Figure 5:**
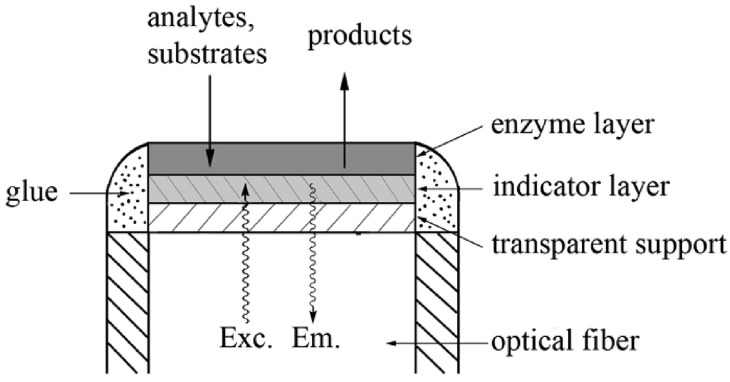
Cross section of a typical fiber-optic enzymatic biosensor. The analyte (substrate) enters the enzyme layer and is converted into products. The indicator (sensing) layer consists of an indicator dye in a polymer layer and registers the formation of reaction products or the consumption of co-reactants such as oxygen. The transparent support is inert and used only to facilitate manufacturing. Exc. and Em. symbolize the paths of exciting and emitted light, respectively. Reprinted with permission from [68]. Copyright 2008 American Chemical Society.

The easiest way to achieve specificity in an optical biosensor is by using more or less specific biorecognition elements such as enzymes, antibodies, oligonucleotides, or whole cells and tissues [68]. Enzymes catalyze reactions with a high degree of specificity, and the products of these reactions (or of reactants consumed) are monitored directly if they are luminescent or colored or by using optical transducers. The steady-state concentration of detectable species is, thus, related to the concentration of the analyte [68]. A cross-section of an optical enzymatic biosensor based on fiber optic comprising an enzyme layer, an oxygen/pH-sensitive indicator layer, and transparent inert support assembled on an optical fiber is shown in Figure 5. The indicator layer is prepared by either dissolving an indicator dye directly in the polymer matrix or covalently or physically adsorbed onto the surface of microbeads, which can be mixed with the polymer matrix afterward. The indicator layers sense the product’s generation of substrate consumption during an enzymatic reaction.

**Figure 6:**
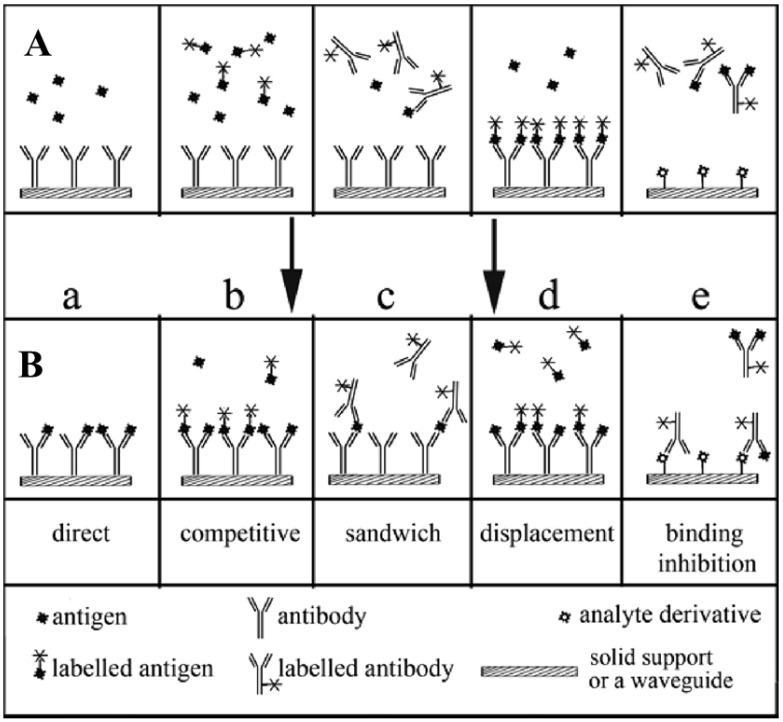
Typical formats of heterogeneous optical immunoassay; situations before (upper part, (**A**)) and after equilibration (lower part, (**B**)). (a) An unlabeled antigen binds to an unlabeled antibody, (b) A labeled antigen binds to an unlabeled antibody, (c) the antigen and fluorescently labeled second antibody are either premixed or added sequentially to the biosensor containing an immobilized primary antibody. (d) involves an initial saturation of all the antibody binding sites with a fluorescently labeled antigen. Upon introduction of the unlabeled antigen, displacement of the labeled antigen occurs and is measured in this sensor as a decrease in the fluorescence intensity. (e) involves the immobilization of an unlabeled analyte derivative on the surface of a waveguide. In the absence of the antigen, the labeled antibody can bind to the surface. Binding is inhibited, however, in the presence of the analyte because it blocks the binding sites (paratopes) of the antibody. In practically all cases, the enzyme-linked immunosorbent assay (ELISA) is a modification of the sandwich method as it uses an enzyme as a label. Thus, a subsequent enzymatic reaction is required to produce a colored or fluorescent product whose concentration can be determined, usually in solution and not on the sensor’s surface. Reprinted with permission from [68]. Copyright 2008 American Chemical Society.

The enzyme(s) can be immobilized onto the surface of the polymer membrane chemically or physically by entrapment into a polymer network such as hydrogels. In Figure 5, a sandwich sensor is mounted on the tip of an optical fiber that transmits excitation light from a light source to the sensor foil and emits (reflected) light back to a photodetector [68]. A variation of the optical sensors that exploit chemiluminescent and bioluminescent reactions is usually more straightforward because no indicator layer is required. They are widely used to monitor highly significant analytes in clinical medicine, food and environmental analysis, and bioprocess monitoring. An affinity-based optical sensor is exemplified using an immunosensors format (Figure 6). In theory, an antigen-antibody binding event is a reversible non-covalent interaction. Still, in practice, most immunoreactions are irreversible due to the significant association constants and very slow dissociation rates [68]. However, efforts have been directed towards the search for reusability of the immunosensors by washing with solutions of high osmolarity, high ionic strength, or low pH, which allows for multiple measurements with a single sensor.

Due to the easy operating principle and quick fabrication of complex 3D models, the additive manufacturing technique has extended to a broad spectrum of biosensing applications, including optical sensors. Three-dimensional (3D) printed structures with integrated sensing components have been widely applied to detect physiological parameters, including blood pressure, heart rate, body motion, respiration rate, brain activity, and skin temperature [74]. Generally, the 3D printed sensors are fabricated by integrating the sensor in the printed platform or directly printing the sensing component.

#### 5.1.2. Electrochemical Biosensor

In an electrochemical biosensor, biological response is converted into an electrical signal for the detection of specific analytes in a wide range of applications, including pathogen detection, disease diagnosis, food safety, and environmental monitoring [75], by integrating the sensitivity of electroanalytical methods with the biological selectivity of the biological component [76]. Like other types of biosensors, electrochemical biosensor typically consists of a biorecognition element that recognizes its analyte, resulting in a catalytic or binding event that ultimately produces an electrical signal that is proportional to the concentration of the analyte, as monitored by a transduction element.

Electrochemical biosensors fall into two main categories—biocatalytic devices and affinity sensors, depending on the nature of the biological recognition processes. Biocatalytic devices incorporate enzymes, whole cells, or tissue slices that recognize the target analyte and produce electroactive species. Affinity sensors rely on a selective binding interaction between the analyte and a biological component, such as an antibody, nucleic acid, or receptor. Biocatalytic biosensors leverage the catalytic power of enzymes to achieve ultra-low detection limits. They are widely used, but because many biochemical analytes are not amenable to enzymatic detection due to a lack of sufficiently selective enzymes, the analytes and affinity biosensors have appeared as an alternative method. Affinity sensors use the selective and robust binding of biomolecules, such as antibodies (Ab), membrane receptors, or oligonucleotides, with a target analyte to produce a measurable electrical signal [76]. The molecular recognition in affinity biosensors is mainly determined by the complementary size and shape of the binding site to the analyte of interest. The high affinity and specificity of the biomolecule for its ligand make these sensors very sensitive and selective [77].

**Figure 7:**
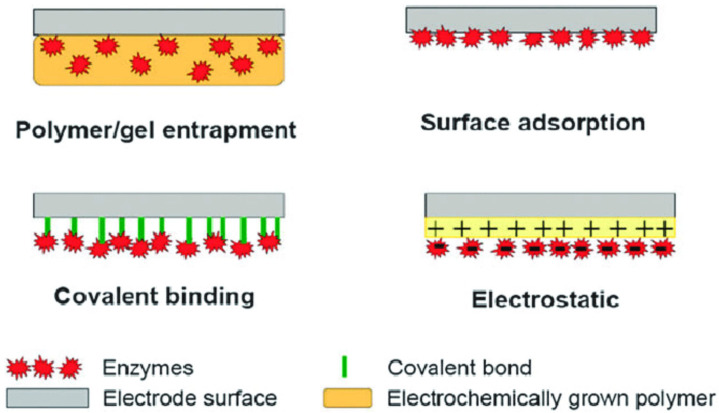
Common methods of immobilizing biomolecules onto an electrode surface. Reprinted from [76], Copyright 2010, RSC.

Many physical and chemical approaches have been studied to immobilize the biomolecules onto the electrochemical transducer to achieve the closest proximity between the biomolecules and the transducer’s surface. A desirable immobilization method would be one that stably retains the structure and function of an immobilized biological entity. Immobilization should be able to achieve enhanced stability, recyclability, and selectivity. Commonly employed immobilization strategies are elucidated by [76], as shown in Figure 7.

The most accessible approach has been physically immobilizing the biomolecules on the electrode surface. Physical immobilization does not involve covalent bond formation; as such, the native structure of the biomolecules is preserved. Chemical immobilization usually involves covalent bond formation between the functional groups (NH_2_, COOH, OH, and SH) biomolecules and the electrodes. Standard enzyme immobilization methods include enzyme entrapment against the electrode using a preformed membrane, encapsulation, inclusion in a gel or electropolymerized film, incorporation in a carbon paste, and using bio-specific interactions such as biotin–avidin binding, adsorption, cross-linking, and covalent attachment (Figure 7) [76,78,79].

Electrochemical techniques are generally organized into three main categories of measurement: current, potential, and impedance. The following electrochemical detection methods—voltammetry/amperometry, electrochemical impedance spectroscopy (EIS), conductometry, and potentiometry—have been employed to varying degrees of popularity depending on the analytical needs.

Electrochemical techniques can significantly benefit from using 3D printing technologies in terms of the relatively lower construction costs of custom-made, complex measurement systems and the great flexibility offered by 3D printing technologies [80]. Specifically, 3D printing can be used to create conductive electrodes with unique shapes or compositions. These electrodes can be utilized for redox and catalytic processes that are useful in electrochemical sensors, presenting a promising avenue for developing novel biosensors [81]. This approach enables the integration of bioelectronics with a three-dimensional environment for conducting biological assays, thereby enhancing the sensitivity and accuracy of the sensor [81,82]. The scalability of this approach further contributes to the production of biosensors with improved capabilities for various applications. For instance, Cantù and colleagues reported the realization of miniaturized sensitive electrochemical platforms for protein detection developed through aerosol jet 3D printing [83]. The authors showed the possibility of improving the reliability and repeatability of measurement techniques integrable in several biotechnological applications using 3D printing technologies.

#### 5.1.3. Physical Sensor

A physical sensor is a device that detects and responds to material inputs, converting them into analog or digital forms. Physical sensors can detect physical quantities based on various physical effects such as force, heat, light, electricity, magnetism, and sound. An example of a physical sensor that involves mass change or calorimetry is the micro-electro-mechanical systems (MEMS) resonant mass sensor. This sensor has been developed to directly measure single adherent cells’ biophysical properties, mass, and growth rate, demonstrating its capability to detect mass changes [84]. An example of a physical sensor is the magnetoelastic sensor. Magnetoelastic sensors have attracted considerable interest within the sensor community as they form an excellent sensor platform that can be used to measure a wide range of environmental parameters, including pressure. Magnetoelastic sensors have been used by applying a mass-changing chemically responsive layer to monitor chemical analyte concentrations, including glucose, carbon dioxide, ammonia, and pH [85]. Coating the magnetoelastic sensor with a mass-changing, chemically responsive layer enables the realization of chemical sensors [85].

Piezoelectric sensors are another class of physical sensors that have found a broad application in biomedical engineering and health monitoring due to their high sensitivity and fast response time. Piezoelectric sensors operate based on the interconversion of electrical and mechanical energies; as such, there has been a growing interest in the use of piezoelectric polymer sensors for energy harvesting and self-powered sensing, leveraging their flexibility, low density, and high piezoelectric constant [86]. This kind of sensor technology demonstrates a high versatility and adaptability towards the fabrication of self-powered and label-free biosensors for the detection of biomarkers, with a wide range of sensing and actuation applications [87,88].

Acoustic wave sensors are among the physical sensors that have emerged as versatile and indispensable tools with applications ranging from biosensing and medical diagnosis to industrial monitoring and safety assurance. These sensors leverage the principles of acoustic wave propagation to detect and analyze physical, chemical, and biological parameters. For instance, surface acoustic wave (SAW) sensors, including Love mode acoustic wave sensors, have been proven to be highly sensitive and reliable for biosensing applications [89,90]. This sensing approach involves the transmission of an acoustic wave across the surface of a device substrate to an interdigitated transducer, where it is converted back into an electric signal through the piezoelectric effect, whereby any alterations to the mechanical wave are reflected in the output electric signal, allowing for the quantification of changes in the surface properties of the device substrate [91,92]. The preceding principle forms the basis for acoustic wave sensors where the SAW is adjustable by adding mass to the surface or by changing the length of the substrate and the spacing between them [91]. Also, film bulk acoustic wave resonators (FBARs) have received specialized attention in electronics and communications for sensing physical parameters such as temperature, pressure, and humidity and for detecting various biochemical substances [93].

### 5.2. Bioprinting Method Applications in Biosensors

Three-dimensional (3D) printing technologies will soon impact the biosensor community at the sensor and sensing layer organization level. Many sensors are intrinsically sophisticated and are often arranged into composite architectures constructed from multiple components [94]. Therefore, new fabrication methods that could be used to create complex sensors rapidly are desired. Many thanks to the rapidly advancing field of additive manufacturing that can enable printing different biomaterials into intricate 2D and 3D architectures, which could be used for sensing.

The convergence of additive manufacturing processes with biomaterials has introduced a new paradigm in the biotechnology engineering community. Indeed, the emergence of new printing materials and a variety of 3D printers for a seamless fabrication of complex hydrogel scaffolds that permit the incorporation of sensing layers within the scaffold with complex geometries have brought new perspectives to most biosensors’ developers [95], whereby 3D bioprinting is now being extended to include critical biotechnological applications such as incorporation of active biomolecular recognition element into the 3D printed objects for (bio)sensing purposes.

Many bioprinting techniques, such as electrodeposition, ink-jet printing, microcontact printing, and extrusion, can be adapted for use in the development of biosensors by a precise, rapid deposition and patterning of the printing material laden with reporter biomolecules. The library of biomolecules that have been bio-printed ranges from biomolecules such as proteins, enzymes [96], nucleic acids, polysaccharides, and bacterial cells to whole cells such as mammalian cells, algae, and bacteria [97].

Three-dimensional (3D) bioprinting of biosensors can benefit from the capability for multiplexing and high-throughput analysis for rapid multianalytes screening. Concerning tissue engineering, the incorporation of sensing capability into 3D printing materials could facilitate a rapid patterning of different sensor molecules over a wide range of concentrations to allow for the detection of threshold levels of biomarkers of cellular responses, thereby allowing for a kind of spatial-temporal monitoring of cellular environment in parallel experiments. The transduction properties of the various biomaterials used as bio-ink are essential when bioprinting is aimed at biosensing applications. The immobilization of BRE is critical in fabricating a sensorized 3D construct. Different fabrication approaches have been employed to date.

### 5.3. Approaches of Introducing Biosensors 3D Bio-Printed Biosensors

**Figure 8:**
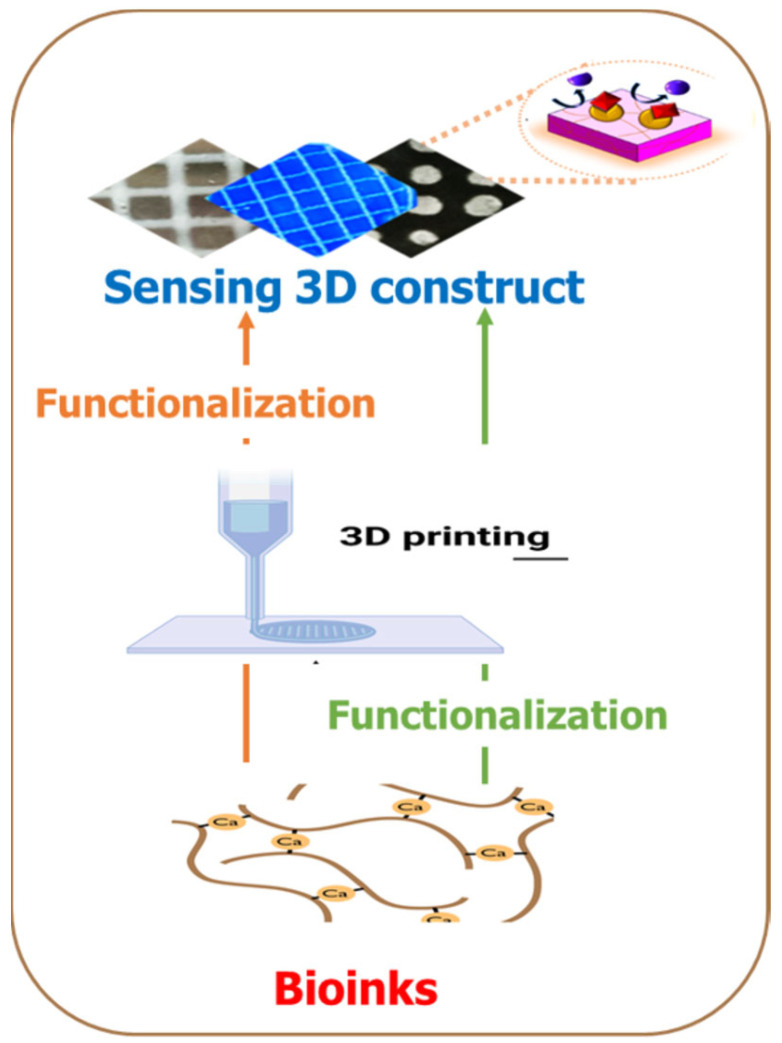
Two strategies for introducing sensing capabilities in additive manufacturing processes. In one approach, the bioink is functionalized with the sensing unit before the 3D printing process, while the second approach entails the functionalization as a kind of post-printing processing.

The ability to rapidly manufacture functional sensors would benefit numerous healthcare and environmental monitoring applications. There are different strategies for introducing sensor units to 3D fabrication. The biomaterials could be functionalized with the BRE during the bio-ink preparation before printing or simultaneously during printing. At the same time, it is also possible to functionalize the 3D construct post-fabrication (Figure 8). As shown in Figure 5, the additive manufacturing process offers the unique ability to seamlessly integrate complementary fabrication processes or subcomponents manufactured using traditional methods. This allows for the fabrication of 3D-printed sensors by embedding a sensor unit directly into the printed structures during a process interruption, or the sensors can be entirely printed as an intrinsic feature of the structure [98]. Integrating sensing into customized complex geometries is beneficial for many applications, such as patient-specific 3D biomedical devices, point-of-care diagnostics, and spatial-temporal monitoring of cellular environment in tissue engineering, to name a few.

**Figure 9:**
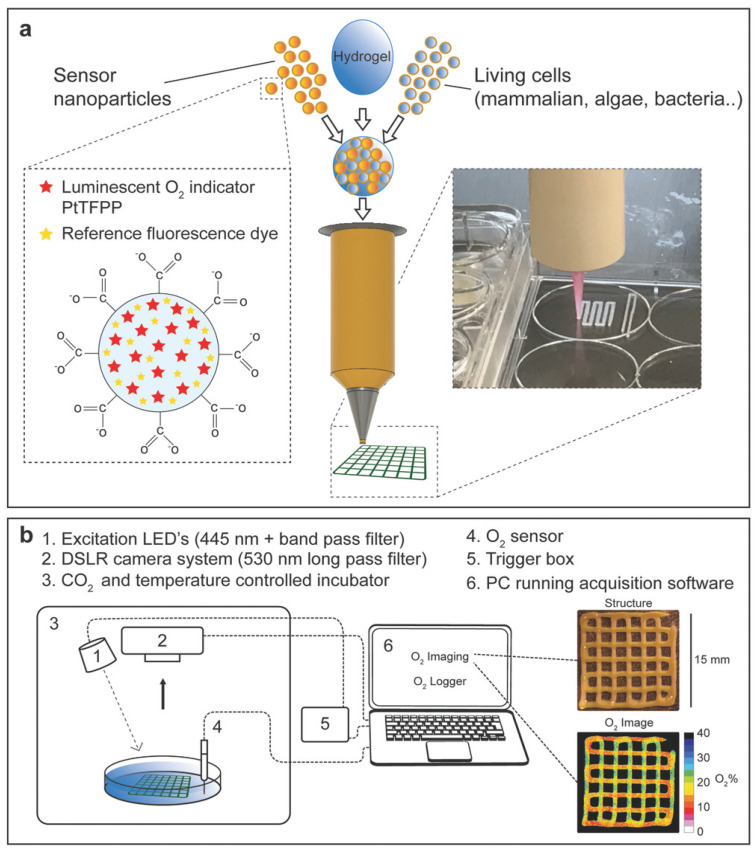
A novel approach for 3D bioprinting with bioink functionalized with sensor nanoparticles. (**a**) Living cells and nanoparticles containing the O_2_-sensitive luminescent indicator platinum (II) meso (2,3,4,5,6-pentafluoro) phenyl porphyrin (PtTFPP) and an inert fluorescent coumarin dye (Bu3Coum) were mixed into an alginate/methylcellulose hydrogel blend for extrusion-based 3D bioprinting. (**b**) Experimental setup for incubation and imaging of structure and O_2_ distribution in 3D-printed hydrogel scaffolds. All components (1–4) besides the LED excitation source trigger box (5) and the PC (6) were placed inside a thermostat incubator with a controlled gas atmosphere. The camera system and excitation LED were placed above the bioprinted constructs placed in a Petri dish with medium, the O_2_ content of which was monitored by an O_2_ optode. Images show the structure of a bioprinted hydrogel scaffold with nanoparticles only (vertical strands) and nanoparticles with microalgae (horizontal strands) and the corresponding O_2_ concentration map. Reprinted with permission from [95] Copyright 2018 Wiley.

In 2018, Trampe et al. demonstrated the possibility of combining 3D printing with incorporated sensor particles into the bio-ink by functionalizing the bioink with luminescent oxygen sensors [95]. The authors developed a simple method to functionalize an alginate-based bioink with luminescent O_2_-sensing nanoparticles, showing excellent printability and biocompatibility when fabricated microalgae and/or mammalian cell-laden scaffolds. The 3D-printed optical sensor was applicable for spatiotemporal imaging of oxygen within the printed—construct to facilitate a rapid evaluation of cell activity in printed constructs as a function of structural complexity, metabolic interactions in mixed-species bio-prints, and response to external incubation conditions. The overall procedure is summarized in Figure 9. However, successfully printing the scaffold with active biomolecular recognition requires optimized temperature and aqueous environment conditions, especially when the recognition molecules of interest are enzymes, antibodies, or other structurally complex macromolecules [2].

**Figure 10:**
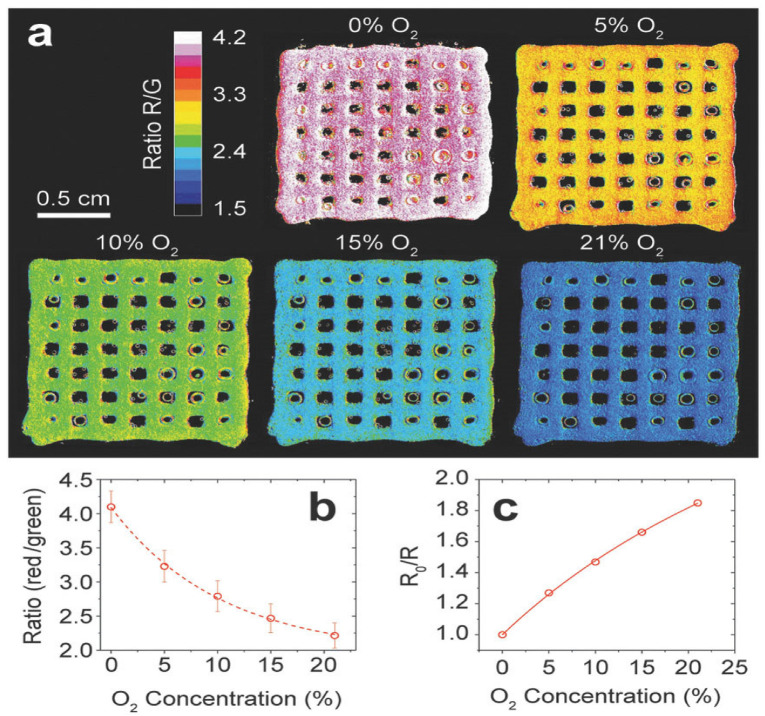
Calibration of a 3D-printed construct composed of hydrogel containing optical O_2_ sensor nanoparticles. (**a**) Ratio images (red channel/green channel) of a printed scaffold with nanoparticles incubated at different O_2_ concentrations. (**b**) Calibration curve obtained from the images in panel (**a**); values represent the mean of the entire scaffold with standard deviation, and the dashed line shows a curve fit of a monoexponentially decay function (r2 > 0.998). (**c**) Stern–Volmer plot of the calibration curve fitted to Equation (r2 > 0.999). Reprinted with permission from [95] Copyright 2018 Wiley.

The O_2_-sensitive nanoparticle-functionalized bioinks were subjected to printability and viability tests, after which the 3Dprinted construct was calibrated according to Figure 10. This extensive calibration experiment showed that the ratio of the red and green channels in the acquired images could describe the O2 dependence of the nanoparticle luminescence in the matrix. Furthermore, the authors observed the absence of nanoparticle leakage from the printed hydrogel scaffold into the surrounding medium during several days of incubation. Moreover, no significant photobleaching was detected under the experimental irradiance levels employed [95].

**Figure 11:**
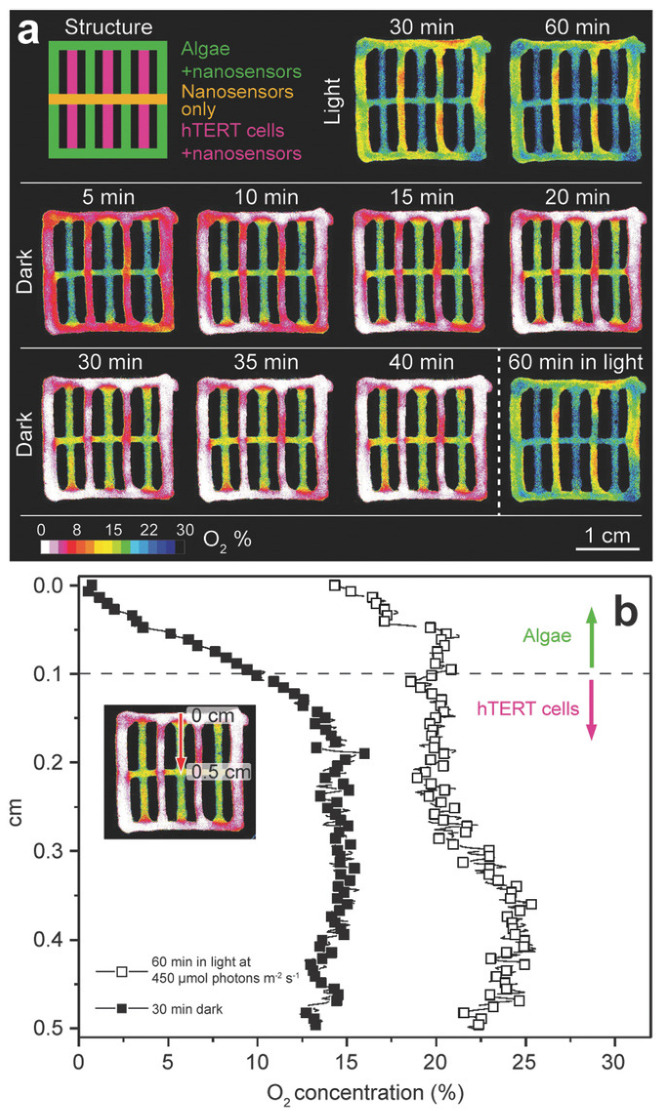
Spatiotemporal dynamics of O_2_ concentration in a multilayered 3D-bioprinted construct composed of hydrogel layers with sensor nanoparticles only, nanoparticles plus microalgae or mammalian hTERT-MSC. (**a**) Visualization of the different scaffold layers and images of O_2_ concentrations in the scaffold after 30- and 60-min exposure to a photon irradiance of 450 µmol photons m^−2^ s^−1^ and as a function of time after subsequent darkening. (**b**) Lateral profiles of O_2_ concentration between the hydrogel layer with microalgae + nanoparticles and a layer of hTERT-MSC + sensor nanoparticles (see red arrow on the inset) measured after 60 min light exposure and 30 min of darkness, respectively. Reprinted with permission from [95] Copyright 2018 Wiley.

Interestingly, the work of Trampe et al. demonstrated the possibility of mapping local differences in O_2_ concentrations due to different metabolic activities in hydrogel compartments with mammalian and microalgal cells. More complex 3D-printed hydrogel scaffolds comprising sensor nanoparticle-laden hydrogel strands with either microalgae, mammalian cells, or without cells were analyzed to demonstrate the ability to map local differences in O_2_ concentrations due to different metabolic activities in hydrogel compartments with mammalian and microalgal cells, respectively Figure 11.

The approach in this study is a landmark and opens a window of opportunities to design unique scaffolds enabling optimal O_2_ supply to mammalian cells growing in the 3D constructs. The ability to conduct online imaging of dynamic changes in O_2_ concentration as a proxy for metabolic activity is a powerful tool in tissue engineering. The use of sensor-functionalized bioinks has a wide range of applications in 3D bioprinting and additive manufacturing, as it enables simple, rapid, and noninvasive mapping of the chemical microenvironment and activity of embedded cells in printed scaffolds [95]. Another sensor molecule, such as a pH-sensitive molecule, can be incorporated in hydrogel-based bioinks, individually or in combination with O2 nanoparticles, in an attempt to create multiparameter measurements and mapping of chemical microenvironments, concentration gradients, and dynamics in 3D-bioprinted constructs with living cells [99].

**Figure 12:**
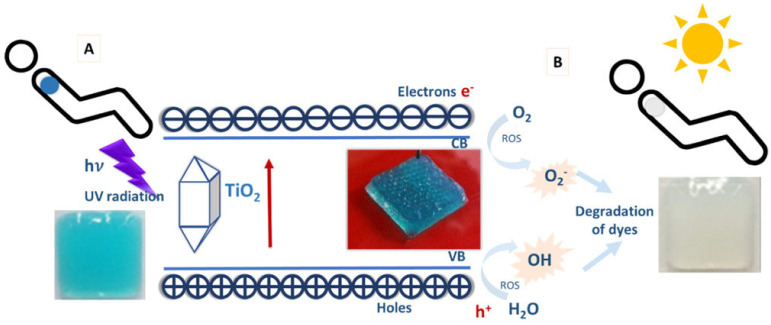
Photocatalytic sensing mechanism used to obtain the colorimetric response employed in the sensor; sensor before UV exposure (**A**) and after UV exposure (**B**). Reprinted with permission from [50] Copyright 2020 American Chemical Society.

Due to the detrimental effects of excessive exposure to ultraviolet (UV) radiation on human health, an affordable, simple sensor for monitoring UV radiation is desirable. In 2020, Finny et al. [50] prepared a hydrogel-based 3D printing ultraviolet (UV) sensor to quantify exposure. In their study, a color-changing hydrogel ink containing alginate, gelatine, photoactive titanium dioxide nanoparticles, and dyes (methyl orange, methylene blue, and malachite green) was first developed and 3D printed [50]. The nanoparticles initiate photocatalytic degradation of dyes, leading to dye discoloration. The authors show that TiO_2_ nanoparticles conserve their photocatalytic properties inside transparent hydrogels, retaining their stability upon exposure to UV light. The sensing mechanism involves the UV-initiated degradation of dyes from intensely colored to colorless, which is summarized in Figure 12.

**Figure 13:**
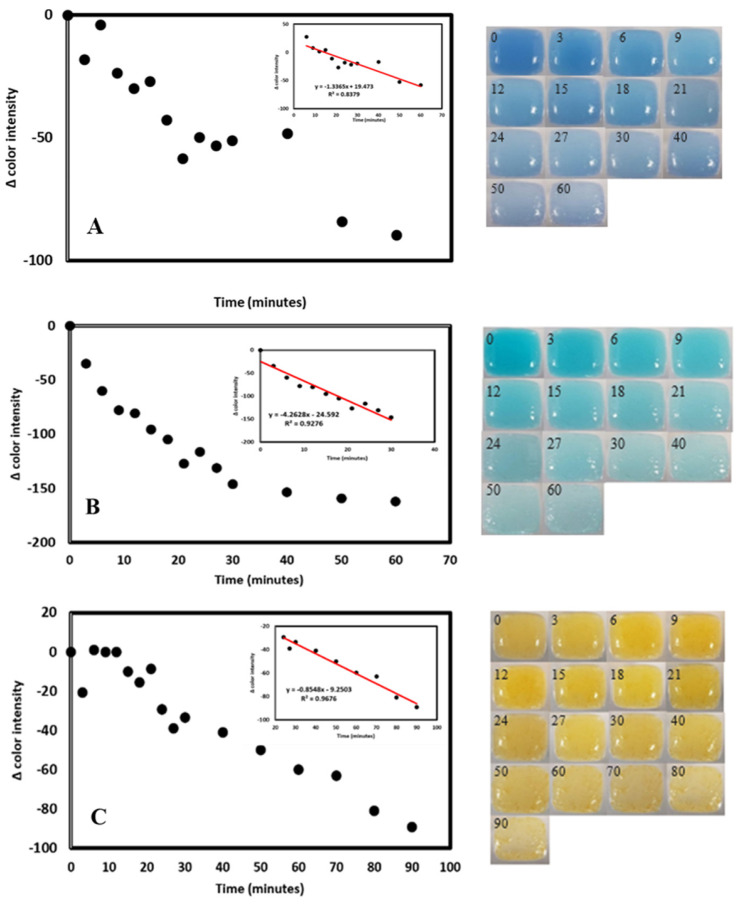
Color discoloration over time with sun exposure. Graphs show a decrease in color intensity of images taken with a cell phone and measured with ImageJ software and pictures of Methylene Blue (**A**), Malachite Green (**B**), and Methyl Orange (**C**) sensors. Reprinted with permission from [50] Copyright 2020 American Chemical Society.

Attempts were made to assess the photo discoloration rate under outdoor exposure conditions and determine the correlation between discoloration and UV exposure. The sensors prepared from the three dyes were exposed to outdoor sun under the recorded weather conditions and UV index. The data presented in Figure 13 illustrates a progressive decline in dye coloration within the printed sensor as time elapses. This finding ascertained the sensor’s functionality and ability to monitor UV exposure levels in the external environment effectively.

The viscosity and composition of the ink were optimized to achieve printability, which resulted in a one-step fabrication approach. The resulting sensors are inexpensive, stable, extremely robust, biodegradable, and easy to use [50]. The hydrogel, comprising alginate and gelatine biopolymers, provided an excellent medium for stabilizing the nanoparticles and the dyes. The hydrogel composition was optimized for room temperature gelation and to facilitate 3D printing of mechanically stable, robust, and reproducible constructs. The ink’s tunability, biocompatibility, and printability offer excellent potential for developing advanced 3D printing methods that, in addition to UV sensors, can be applied more broadly to fabricate other sensing technologies for various other applications. It is worth noting that the sensors produced in this study are low-cost and highly scalable, making them an attractive option for developing other sensing technologies. The optimized ink formulation and printing conditions can serve as a valuable platform for generating 3D-printable constructs for different applications.

Worthy of mention is the work of Liu and Li [100], who reported a 3D printing-based strain sensor using an ultra-stretchable and self-healing double-network hydrogel. Leveraging the thermoreversible sol–gel transition behavior of κ-carrageenan in water, a double-network (DN) hydrogel was prepared by combining an ionically cross-linked κ-carrageenan network with a covalently cross-linked polyacrylamide (PAAm) network, showing an enhanced self-healing feature and excellent recoverability. The warm pre-gel solution of the dual network was 3D printable.

**Figure 14:**
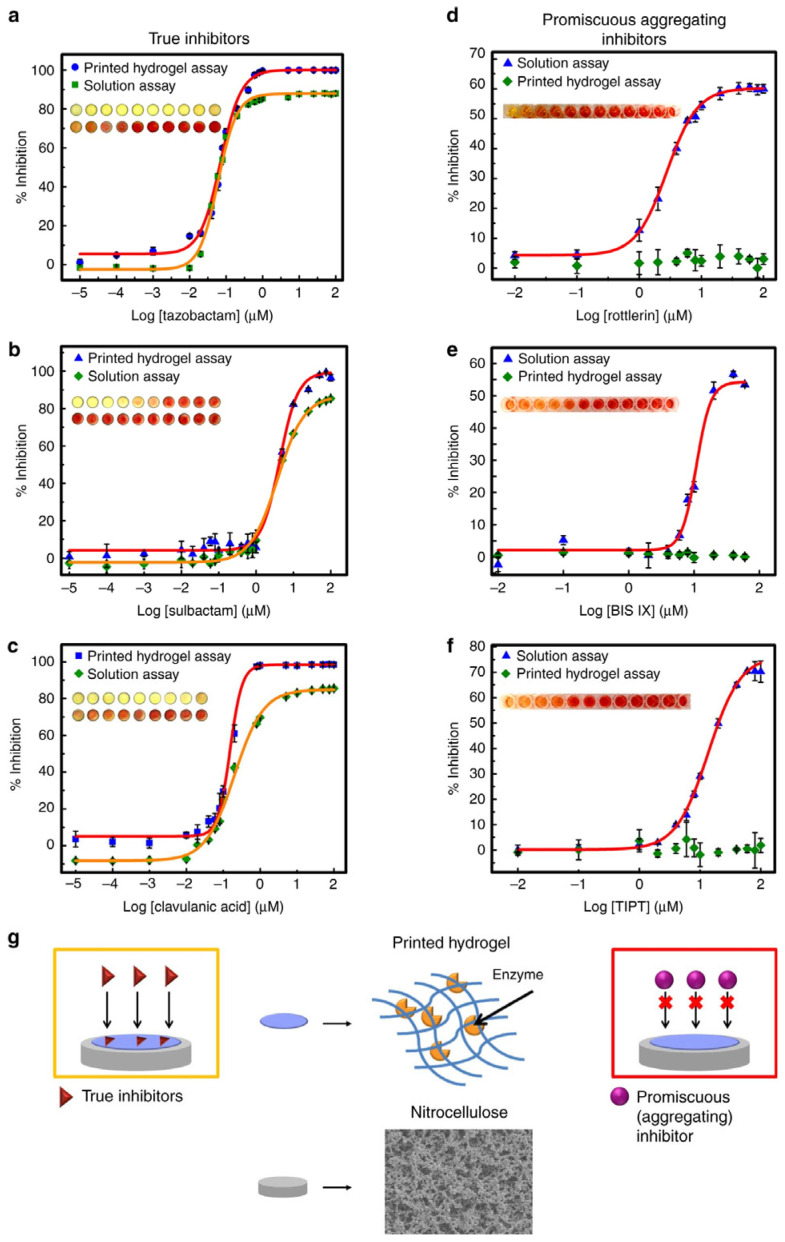
A printed hydrogel-based β-lactamase drug-screening assay. (**a**–**c**) The printed hydrogel-based β-lactamase screening assay can determine dose–response relationships of classic β-lactamase inhibitors. Comparison of the solution vs. printed hydrogel-based inhibition curves for true β-lactamase inhibitors: tazobactam; (**b**) sulbactam; (**c**) clavulanic acid. (**d**–**f**) The printed hydrogel-based β-lactamase-screening assay can discriminate between true and promiscuous aggregating inhibitors. Comparison of solution vs. printed hydrogel-based inhibition curves for known promiscuous inhibitors of β-lactamase: (**d**) rottlerin; (**e**) BIS IX; (**f**) TIPT. (**g**) Schematic illustrating how the size selectivity of the printed hydrogel layer excludes the colloid-forming drug (promiscuous aggregating inhibitor) from accessing the encapsulated enzyme but can permit the diffusion of a soluble drug (true inhibitor) to generate a positive signal for true inhibitors only. Data are presented as means ± SDs (*n* = 3). Reprinted from [101].

Also, a printable hydrogel microarray-based drug-screening platform capable of unambiguously differentiating true enzyme inhibitors from false inhibitors has been developed, by immobilizing the enzyme through entrapment within the hydrogel [101]. A drop-on-demand syringe solenoid printer was used to print hydrazide (POH) and aldehyde sequentially (POA) functionalized poly(oligoethylene glycol methacrylate) (PO) precursor polymers, previously shown to rapidly gel upon mixing via hydrazone bond formation [102] on a nitrocellulose substrate. To demonstrate the potential of printable hydrogel-enzyme thin films for practical biosensing applications, TEM-1 β-lactamase was printed in a POA/POH hydrogel array onto the microzones of a 96-well nitrocellulose wax-printed template. This process created a microplate mimic adaptable to current high-throughput screening protocols, as depicted in Figure 14 [101]. Inhibitor solutions and nitrocefin (a colorimetric β-lac substrate) were deposited on the microzones at different concentrations using a high-throughput dispensing robot. The resulting colorimetric read-out of β-lac activity was quantified via image analysis [101]. This report elucidates a novel technological advancement in drug discovery that employs a printed hydrogel screening assay instead of the conventional microplate assay platforms. This innovative method circumvents the primary limitations of the existing microplate assay platforms by substantially reducing reagent volumes, negating the costs associated with microtiter plates, and augmenting the assay’s sensitivity.

**Figure 15:**
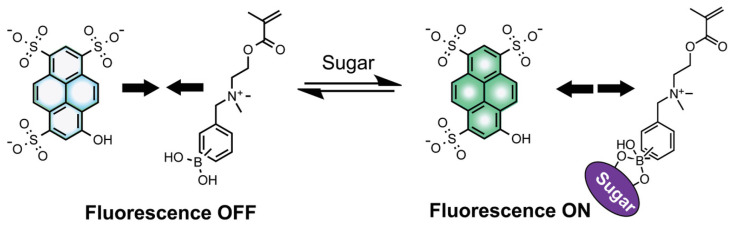
Working principle of a two-component sugar sensing system composed of the fluorophore pyranine and the BA monomers, *N*-(2-boronobenzyl)-2-(methacryloyl-oxy)-*N*, *N*-dimethylethane-1-ammonium bromide (*o*BA) or *N*-(3-boronobenzyl)-2-(methacryloyloxy)-*N*, *N*-dimethylethane-1-ammonium bromide (*m*BA), used in this study. Reprinted with permission from [103] Copyright 2020 Wiley.

Recently, a gelatine methacrylamide-based hydrogel harboring a sugar-sensitive fluorophore has been printed as a 3D sugar-sensing hydrogel [103]. This work was based on the possibility of fluorescently monitoring the reversible binding ability of boronic acids (BAs) with diols such as sugars. The author reported an extrusion-based 3D-printed sugar-sensing hydrogels by incorporating a boronic acid–fluorophore (BA-fluorophore) pair in a gelatin methacrylamide-based matrix. The principle behind the sensing system is based on the intermolecular interaction between BA and fluorophore, resulting in a quenched state of fluorescence [103]. But in the presence of sugar, cyclic BA-ester forms, which can induce a structural change around boron, thus weakening the interaction between the BA unit and fluorophore, and thereby restoring the fluorescence. (Figure 15) [103]. In so doing, saccharide concentration can quantitatively modulate the fluorescence response.

**Figure 16:**
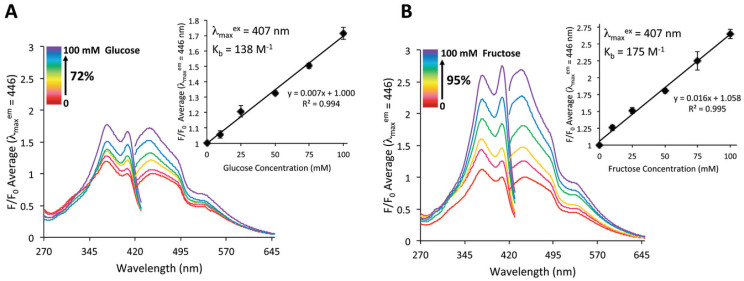
Fluorescence response of the hydrogel scaffolds in 0–100 mM glucose (**A**) and 0–100 mM fructose (**B**) at pH 7.4, where *F*_0_ is the initial fluorescence of the scaffolds in pH 7.4 buffer, and *F* is the fluorescence after the sugar addition. The excitation wavelength was 407 nm, and the corresponding emission wavelength was 446 nm. The points on the curve represent the mean ± the standard deviation (*n* = 3). Reprinted with permission from [103] Copyright 2020 Wiley.

Accurate and reproducible fluorescent detection of saccharides over physiologically relevant concentration ranges (up to 40 mM) has been demonstrated. The hydrogel fluorescence emission increases linearly in the presence of glucose (1.72-fold) or fructose (2.65-fold) up to 100 mM (Figure 16).

Mandon et al., 3D printed objects with entrapped sequential enzymatic reactions (glucose oxidase and peroxidase) and entrapped the antibody for a sandwich immunoassay to detect brain natriuretic peptide [2].

Most recently, Leggett and colleagues [23] 3D printed pH-indicating filaments of poly-lactic acid by using a fused filament fabrication (FFF) approach. In this study, polylactic acid (PLA) and poly-(ethylene glycol) (PEG) were blended with pH indicator powder to prepare filaments with environmental sensing functionalities. The novel PLA-PEG-indicator sensor filament was robust, with characteristic color changes in different pH conditions tested, thermally stable, and biodegradable. The fabrication approach entailed pre-mixing the components—PLA, PEG, and the indicators (bromothymol blue, phenolphthalein, and thymol blue) before extrusion. A particular type of extrusion-based 3D printing—the direct ink-write technique—was employed to additively manufacture complex geometrical structures with an embedded wireless temperature and relative humidity (RH) sensor during the 3D-printing process [65]. The printed sensor object could read up to 65 RH and temperatures of up to 85 °F from a maximum distance of 141.7 m. This work revealed the feasibility of creating complex geometrical shapes with ceramic using the DIW printing technique [65]. This is one of the pioneering studies demonstrating the possibility of integrating functional capabilities into 3D-printed ceramic objects, exemplified using temperature and humidity sensor functionalities.

A reagent-less additively manufactured sensor for multi-analytes has been developed by Finny and colleagues [104]. The hydrogel-based (bio)sensors with incorporated receptor molecules and transduction interfaces were 3D printed by extruding the bioink formulation comprising enzymes and catalytic and photoactive properties. The 3D-printed biosensors were a lactate sensor for measuring physiological activity in the sweat and a UV sensor for quantifying harmful UV radiation exposure.

The facile integration of chemical sensing technologies into 3D-fabricated manifolds, which can garner quantitative, measurable responses to the local environment, remains an unmet need of tissue engineering. For example, the incorporation of sensing units into a hydrogel for glucose detection in cancer cells [32] and tissue culture [33,34] has been reported. Glucose detection in solution has been well documented using boronic acid (BA) that binds reversibly to diols of glucose and fructose, leading to a quantitative fluorescent response. A transition from solution-based methodologies to a solid, insoluble platform must be made for the practical realization of this technology. Therefore, Bruen and colleagues [103] researched how to create a 3D-printed hydrogel-based sugar sensor. BA–fluorophore pair was incorporated into a gelatin methacrylamide-based matrix and fabricated by extrusion-assisted 3D printing. The resulting extruded structured porous hydrogels displayed a measurable and reproducible linear fluorescence response to glucose and fructose up to 100 mM. This is a landmark attempt to generate a 3D-printed structure with chemical sensing capability, and as such, could provide a viable route for applying spatiotemporal sensing capabilities to emerging 3D cell culturing environments [103].

## 6. Future Perspectives

Additive manufacturing allows for the fabrication of functional materials with complex architectures, controlled microstructures, and material combinations. It has continued to influence the fields of biomedical sciences and biotechnology, including biosensors. The advancement of biomedical sensors has been driven by innovations in device manufacturing techniques at the nano, micro, and macroscale. Over the past few decades, biosensor technology has progressed in miniaturization, elasticity, and customization. The need for miniaturization is ever-increasing for the processor and packaging, leading to the fabrication of smaller devices with improved functionality [105] and multiplexing [106]. Despite the constant advancement of biomedical devices, these trends remain consistent, spurring new and more efficient ways of manufacturing device elements. These advances are still in their developmental stage, and several new 3D-printing methods are in the pipeline [107].

The advances in 3D printing technology that enable new capabilities and functionality in biosensors include multi-material printing and simultaneous printing of hydrogels with biomolecular entities using multi-length scale printing and scale-up [7]. Three-dimensional (3D) printers with multiple printheads/nozzles are being developed to scale production rates for various applications, including biosensors. The advancements address 3D printing limitations like post-processing compatibility, layer misalignment, over-extrusion, and anisotropic strength [7,108]. Biomedical sensors in 3D printing are advanced manufacturing applications which enable the creation of complex geometries and customized designs with rapid prototyping and multi-material integration capabilities. Three-dimensional (3D) printing has thus become a widely adopted fabrication approach for various analytical instrumentations. As such, a significant push exists to explore how 3D printing can enhance analytical methodologies. Multiple fields actively research new and complex designs for microfluidics, prototype scale-up novel structures for electrochemical and optical components manufacturing, biopolymers, and functional bioinks using 3D printing [109]. These research areas indicate the potential for future rapid advancements in additive manufacturing applications in biosensors. Regarding electrochemical detection of biomolecules/biomarkers using printed electronics, the main trends in the most updated research refers to the attempt to introduce nanomaterials, such as carbon nanotubes [110], to improve sensitivity, new techniques such as imprinted polymers or specific functionalization to enhance the selectivity [111], and high-resolution printing techniques to improve the control over reproducibility [112].

The ability to functionalize the bioink with biorecognition elements such as functional proteins (enzyme, antibody), DNA, carbohydrates, or biocompatible organic dyes during the additive manufacturing process will open new horizons for introducing sensing capabilities into biotechnological processes such as tissue engineering. This is because non-invasive monitoring of cells’ growth, distribution, and metabolic activity in bio-printed 3D constructs is a growing need yet to be actualized. However, integrating sensing capabilities into tissue engineering protocols via 3D printing technologies requires high repeatability, fast response time, and excellent biocompatibility for live cells.

Because most 3D printing materials and the pre- or post-printing treatments of the construct are not compatible with cell viability requirements, the desire for printing materials with excellent biocompatibility and cell adhesion and requiring less processing is on the rise. Most naturally occurring polymers exhibit appreciable cytocompatibility but lack the structural strength to maintain the shape fidelity of the printed construct post-printing, necessitating the use of composite bioink where one component could serve as a sacrificial ink and the fabrication into solidifying support bath [113]. Sacrificial ink or composites have been successfully used in the 3D printing cell-laden bioinks in tissue engineering protocols [114]. Still, when an additional layer of functionality (such as sensing) is to be incorporated, it is desirable to have as few preparation steps as possible. Thus, chemical functionalization of the biomaterials used in bioink preparation holds a high prospect for seamless integration biomolecular recognition elements and sensing capabilities into the plotted constructs. For example, covalent modification of alginate with reactive chemical groups has been used in optical sensor development by modifying alginate with pyrrole or streptavidin [115,116,117]. As an example of non-conductive and biocompatible natural polymers, alginate is a promising material for biofunctionalization in sensor development because of its chemical modifiability. This biopolymer has been engineered to impart cell adhesion property (using a tripeptide—arginine-glycine-aspartic acid, RGD) [114], photoactivity (by methacrylation) [118] and electrical conductivity using polypyrrole [119], affording it a wide applications in tissue culture and 3D bioprinting. Alginate-streptavidin conjugate can be used as bioink for post-printing functionalization with biotin-containing biomolecules. In addition to the discourse above, the discovery of novel printing materials and the engineering of biomaterials to incorporate functional groups amenable to facile conjugation chemistry offer immense potential to revolutionize the additive manufacturing process in developing biosensors. Additive manufacturing processes are expected to boom due to the discovery of new biocompatible printing materials. This will significantly impact biotechnological applications, including the development of soft hydrogel-based 3D printed biosensor technology.

## Figures and Tables

**Table 1:** Different printing technologies for various applications.

Polymer (Composite)	Source	3D Printing Method	Application	Reference
Alginate dialdehyde-gelatine	Semi-synthetic	Extrusion	Tissue engineering	[63]
poly(lactic acid)/poly(ethylene glycol)	Semi-synthetic	fused filament fabrication (FFF)	pH-sensor	[64]
Gelatine-alginate	Natural	Extrusion	Printability optimization	[59]
Ceramic clay	Natural	Direct ink-writing (DIW)	Temperature and humidity sensor	[65]
Alginate/ZnO nanoparticles	Natural	Extrusion	Wound healing	[66]
Alginate/gelatine/TiO_2_	Natural	Extrusion	UV sensor	[50]

## Data Availability

Data sharing does not apply to this article.
